# Global Warming’s Six MTurks: A Secondary Analysis of a US-Based Online Crowdsourcing Market

**DOI:** 10.3390/ijerph19148320

**Published:** 2022-07-07

**Authors:** Erika Austhof, Heidi E. Brown

**Affiliations:** Department of Epidemiology and Biostatistics, Mel and Enid Zuckerman College of Public Health, University of Arizona, Tucson, AZ 85721, USA; heidibrown@email.arizona.edu

**Keywords:** global warming, audience segmentation, climate change, health communication, public health, risk perception

## Abstract

Using a global warming audience segmentation tool (Six Americas Super Short Survey (SASSY)) as a case study, we consider how public health can use consumer panels and online crowdsourcing markets (OCMs) in research. Through a secondary analysis, we aim to understand how consumer panels and OCMs are similar to or different from each other on demographics and global warming beliefs through SASSY, and how they compare to US Census estimates. With this information, researchers will understand public opinion of global warming in their sample, which is useful for many climate change initiatives. Neither the consumer panel (Ipsos) or OCM sample (MTurk) matched US estimates of population demographics. Both panels achieved similar SASSY segments, showing that even with diverse sampling frames, SASSY is a useful tool for understanding global warming sentiment. Compared to Ipsos, MTurk was younger (more Millennials and Generation X), had higher educational attainment, and lower income. Both panels were majority White, but Ipsos was more diverse than the unweighted MTurk. Ipsos had more respondents from the South whereas MTurk had more respondents from the West. Across the MTurk SASSY segment, there were no significant differences for the majority of demographic characteristics except for age; younger generations were more Alarmed or Concerned, and older generations were more Doubtful and Dismissive. Researchers interested in understanding their sample’s opinions of global warming should use SASSY and consider oversampling in key demographic variables if they intend to achieve a nationally representative and diverse sample.

## 1. Introduction

The Yale Project on Climate Change Communication and the George Mason University (GMU) Center for Climate Change Communication have, since 2008, provided reports and publications on how American’s perceive and react to global warming. American public opinion about climate change and the understanding that global warming is happening and is human-caused reached an all-time high in 2020 [1]. The effects of global warming will have considerable impacts on human health through extreme weather events, decreased air quality, and increased temperatures [2], and most Americans believe global warming can be harmful to health [3,4,5]. The results from these reports and publications have provided a significant contribution to our understanding of how Americans view climate change over time. The authors have encouraged other researchers and groups to replicate their methods in different samples [3], but to our knowledge, there have been few replications of their work outside of the original sample [6], and none in other sampling frames in the US. It is important to determine if public opinion of global warming is similar or different, depending on the sampling frame and methodology used, so that communications and discussion of global warming can be tailored to different audiences.

A representative sample is a subset of a population that seeks to reflect the characteristics of a larger group. Because it is largely impossible to survey every person in a larger population, samples are useful because they contain smaller, more manageable data on a larger group. However, representative samples can be difficult to achieve in public health research [7]. Sampling methods fall into either probability- or non-probability-based methods [8]. Probability sampling means that every member of the population has a known probability of being selected for a given study. In non-probability sampling the sample selected for the study differs from the population, and the degree to which they differ is unknown. Probability-based sampling in public health includes random sampling and stratified but systematic sampling; the most likely sample being the US Census.

Many non-probability-based survey methods are available to public health researchers, including convenience sampling through web-based survey platforms, such as consumer panels, or online crowdsourcing markets (OCMs). We aim to understand how consumer panels and OCMs are similar to or different from each other on demographics and global warming beliefs, and how they compare to US Census estimates. In this secondary analysis, we start by reviewing the similarities and differences between consumer panels and OCMs. Second, we demonstrate through a case study how an audience segmentation tool can be used in an OCM to understand a sample’s opinions of global warming. Finally, we compare demographics across both samples to US Census demographic estimates to understand differences between sampling frames and provide recommendations for future research. With this information, researchers will understand their sample’s opinion on global warming, which they can use for a range of climate change initiatives, including communications, messaging, and research efforts.

## 2. Materials and Methods

### 2.1. Consumer Panels and Online Crowdsourcing Markets (OCMs)

Online survey companies, such as Qualtrics and Ipsos, are market research companies that offer access to consumer panels for research and marketing activities. Consumer panels provide researchers with an able and willing pool of respondents for research, with little effort on the researchers to collect each response. However, they can be costly, and consumer panels may differ demographically from the underlying population unless sampling weights are used. OCMs such as Amazon’s Mechanical Turk (MTurk) [9] provide an alternative to researchers hoping to quickly collect survey responses from a sample. The largest motivating factor for using OCMs is the reduced cost and rapid response time. Buhrmester et al. [10] found that low compensation rates (USD $0.02 to $0.50, survey time 5–30 min) does not affect data quality but it may impact data collection speed (time to complete all needed sample surveys). This low cost provides an added benefit to researchers with limited funds hoping to use MTurk for sampling. Researchers can choose any level of compensation when setting up their survey. While compensation is incredibly low in MTurk, compensation level does not appear to impact construct measurements; researchers still need to include attention checks and use increased scrutiny of the responses to detect workers with low attention or those using automated methods [11].

MTurk participants are slightly more demographically diverse than standard internet samples, and the data obtained is as reliable as other traditional methods [10]. US-based OCM samples also provide similar reliability, convergent validity, and divergent validity [12]. Consumer panels often benefit from having a dedicated pool of respondents, and they can integrate sampling weights for specific demographics if researchers need to over-sample by certain attributes. MTurk and other OCMs also provide an equivalent of sampling weights, called premium qualifications, but at a cost per respondent (range $0.05–$1.00). Therefore, using a consumer panel company versus MTurk may be equivalent in cost if researchers require a very specific sample or perspectives.

Non-probability-based sampling methods, such as consumer panels and OCMs, risk having selection bias and non-response bias [11]. In the case of OCMs, some participants may self-select to take a survey because of their interests in a given topic, and thus they may have more knowledge of a given area than a similar respondent without interest in the topic. Researchers could choose to keep descriptions of their study vague or use waves of recruitment with different descriptions to reduce selection bias [7]. After identifying the sample size required for the analysis, every effort should be made to ensure that an adequate number of respondents complete the survey to reduce non-response bias [7]. If specific populations or quotas are needed to achieve a representative sample, then researchers should pre-specify those quotas using premium qualifications in MTurk or targeted recruitment in consumer panels [11]. In doing so, researchers ensure their respondents do not differ in meaningful ways from non-responders and their underlying population. Finally, when using quota sampling, researchers should ensure they receive sampling weights in order to integrate into their statistical analyses.

### 2.2. Case Study Methods

In 2020, we sought to understand how consumer panel and OCM sampling frames differed by using a pre-validated questionnaire, called the Six Americas Super Short Survey (SASSY) [13,14], which seeks to understand how respondents view global warming. This segmentation tool is designed to quickly understand where a sample falls in their views of global warming by answering 4 questions: (1) “How important is the issue of global warming to you personally” (2) “How worried are you about global warming?”; (3) “How much do you think global warming will harm you personally?”; and (4) “How much do you think global warming will harm future generations of people?” After answering each question, a weight, dependent on the answer provided, is applied and then summed, to determine which segment the respondent falls into [13,14].

The survey we developed for use in MTurk included 67 survey questions from SASSY, demographics, and questions regarding the health impacts of global warming [1]. Demographic questions included gender identity (male, female, transgender male, transgender female, non-binary or gender neutral, and prefer to self-describe (open text)), race and ethnicity (multiple-select White or Caucasian, Black or African American, American Indian or Alaska Native, Asian or Pacific Islander, Hispanic or Latino, and prefer to self-describe (open text)), state, zip code, neighborhood type (urban, town, rural, farm), income (total household before taxes in $10,000 increments), highest education level, and political ideology (recent voting behavior Yes/No, party affiliation, political ideology scale 1–7). We have provided the subset of survey questions we used in this analysis in Supplemental Material, Table S1.

We formatted the survey flow to include attention check questions in each block as described in Smith et al. [15] to encourage data quality and completeness. Data from George Mason University’s (GMU) 2020 distribution of their survey through a consumer panel, Ipsos [1], and the 2020 US Census demographics [16] were compiled for comparison survey datasets. We collected frequencies and proportions from the April 2020 GMU summary report, [1] as well as from the US Census websites [16]. In the 2020 survey (*n* = 1029 in Ipsos), Leiserowitz et al. [1] found consistent results with the previous year’s survey, showing that a record proportion of Americans think global warming is happening (73%) and that global warming is mostly human-caused (62%). Many Americans also believed that they will personally be harmed by global warming (43%) and that a variety of health harms (both physical and psychological) will affect their community as a result of global warming.

The MTurk survey was distributed from 14 to 15 July 2020 for target recruitment of *n* = 200 and from 15 July to 14 August 2020 for target recruitment of *n* = 300. Participants consented to participate in the survey and confirmed that they were at least 18 years of age. Because respondents in non-US OCM samples can significantly vary in their demographics compared to US OCM respondents [12], and to follow the methods of GMU’s sampling restrictions to the US, we restricted our respondents to US respondents only. The survey was self-administered and completed in a web-based environment through Qualtrics. Participants were paid $2 with an estimated 12-min survey completion time. Only MTurk Master’s users were eligible to participate in the first 90% of total survey responses before it was opened up to any MTurk user to assist with the enrollment target of 500 participants. Additionally, at 90% completion, the survey title and description were edited within MTurk to remove any instances of the words “climate change” in order to reduce selection and coverage biases. A sensitivity analysis using chi-squared tests was completed between the first 90% and the remaining survey participants to assess any differences between the participants. We determined that a sample size of 500 respondents would result in a representative sample for this study and was the largest sample we could obtain given resource constraints. We did not include additional premium qualifications in order to determine what the “raw” MTurk base comprised compared to other panels.

### 2.3. Data Cleaning and Analysis

Demographics from our survey (MTurk) and the US Census demographics were mapped to match Ipsos’ demographic variables in order to make comparisons across samples (see Supplemental Material, Table S2). We excluded the iGen age category since we restricted recruitment to adults over 18 years of age. The top segment for each MTurk participant was calculated using the online SASSY segmentation tool [13]. Chi-squared or Fisher’s exact tests (as appropriate) were used to test if the survey samples and responses were significantly different between MTurk, Ipsos, and the US Census. Summary statistics, with standard deviations or confidence intervals where appropriate, are provided at the alpha = 0.05 level. We do not report *p*-values when cells had less than 10 respondents in order to limit generalizations about the results. All data analysis was completed in Stata 16 [17].

## 3. Results

We collected 508 responses from 14 to 15 July 2020 and from 15 July to 14 August 2020. The sensitivity analysis between the first and second waves, as well as between the first 90% and last 10%, did not show any differences. We excluded 4 responses due to failure to answer attention check questions, for a total of 504 responses for the MTurk survey. Compared to the US Census, Ipsos and MTurk were significantly different on the majority of demographic levels (Table 1).

**Figure 1:**
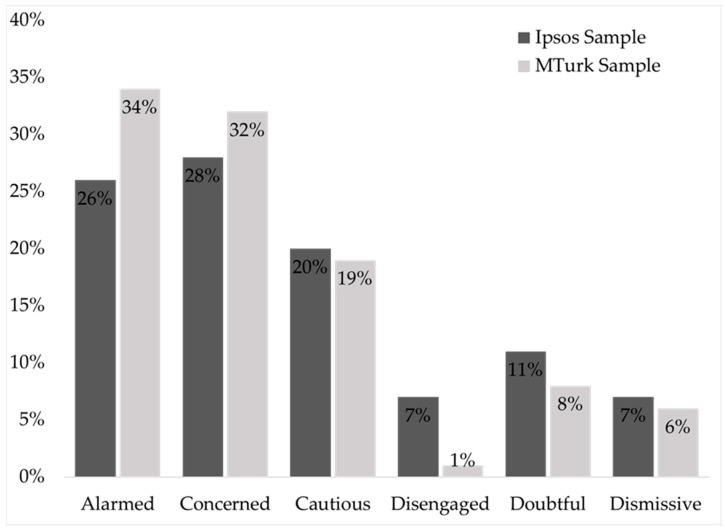
Ipsos National Estimates, April 2020 vs. MTurk Group Data, July–August 2020.

Compared to Ipsos, MTurk was younger (more Millennials and Generation X *p* < 0.001), whereas Ipsos was older (more Baby Boomers and Silent Generation *p* < 0.001) (Table 1). Ipsos had less educational attainment than MTurk, whereas MTurk had a higher proportion with Bachelor’s degree or higher (*p* < 0.001). Although they had higher educational attainment, MTurk had a higher proportion of respondents with lower income levels (less than $75,000 *p* < 0.001), and Ipsos had more respondents with income levels greater than $125,000 (*p* < 0.001). Both panels were majority White, but Ipsos had more Hispanic, multiple race categories, and other race categories than MTurk (*p* < 0.001). There were no significant differences among the samples for categories of gender. Compared to MTurk, Ipsos had more respondents in the South whereas MTurk had more respondents from the West. For the SASSY Segment, MTurk had more respondents in the Alarmed category, whereas Ipsos had more respondents in the Disengaged category (Figure 1).

Across mTurk SASSY segments, there were no significant differences for the majority of demographic characteristics (Table 2). There were significant differences among age categories and SASSY segment (*p* = 0.005); younger generations were more Alarmed or Concerned, and older generations were more Doubtful and Dismissive.

## 4. Discussion

This study provided an overview of the similarities and differences between US-based OCM and consumer panels in order to understand public opinion on global warming using SASSY. One of the main motivations for this work is to show how SASSY can be used to quickly and effectively understand a sample’s opinions about global warming. To our knowledge, SASSY has been used only within Ipsos, even though Leiserowitz et al. [18] have encouraged other researchers and groups to replicate the methods and use of SASSY to understand differences between samples. Yale and George Mason universities have used SASSY, as well as other validated questionnaires and surveys, to understand how American’s view climate change in relation to other areas such as the human health impacts of climate change. A recent survey by Leiserowitz et al. [1] found that over half of Americans view global warming as a public health issue, and Myers et al. [19] found that framing climate change with this lens is most likely to produce behavioral change for climate change adaptation and mitigation. Using SASSY to understanding public opinion on climate change can help with a range of climate change initiatives, and we encourage others to use SASSY in order to determine global warming beliefs of a sample.

In this study, we found that Ipsos and MTurk significantly differed from each other on the majority of demographic variables but had a similar distribution of SASSY segments. This finding shows that even with diverse sampling frames, SASSY is a good tool for understanding sentiments towards global warming. While Ipsos had about equal regional coverage, MTurk appeared to have more in the West and less in the South than Ipsos. In addition to the geography and high levels of poverty compared to the US, Kearney and Bell [20] show the southeastern US is particularly vulnerable to the health impacts of climate change, and the perception of global warming in the poorest counties in the area is that respondents are significantly less likely to believe global warming is happening. The MTurk sample was younger, more educated, and lived more in the West than Ipsos, and, while we found no differences among the Doubtful and Dismissive categories, the Alarmed category was significantly different (34.1%) from Ipsos (26%). While demographically different, both sampling frames achieved similar SASSY segments. Within MTurk alone, there were no significant differences among demographic characteristics, except for age. Older generations were less Alarmed or Concerned. Even though the elderly population is particularly vulnerable to the effects of extreme heat, risk perception of extreme heat in elderly populations is low [21,22,23,24,25]. Demographics alone do not explain global warming beliefs; we encourage the use of SASSY in MTurk as a validated tool to understand beliefs about global warming, rather than focusing on demographics.

Neither consumer panel nor OCM sample matched the underlying US population through the US Census 2020 estimates. Overall, MTurk was younger, more educated, and had higher educational attainment but lower income compared to the US Census, whereas Ipsos was older and less educated but had higher income compared to the US Census. Howe et al. [26] has shown that census tracts with high minority populations, higher populated urban census tracts, and census tracts with higher social vulnerability tend to have higher risk perceptions about extreme heat health impacts, compared to White, suburban/rural, and higher income census tracts. Thus, areas that may be the most vulnerable to climate change impacts may also have the lowest risk perceptions of global warming. MTurk allows for system qualifications of location (including country and state) if researchers aim to investigate specific states or locations. Taken together with our results, this speaks to the importance of targeting public health communications to audiences in vulnerable areas in the US [3,27]. Researchers interested in obtaining a national sample representative of the US Census by using MTurk should over-sample in the South and in older populations. Additionally, MTurk provides access to premium qualifications (for a fee) on age, gender, income, education, and political affiliation, but not race and ethnicity. Through the use of premium qualifications in MTurk, or sampling weights in consumer panels, researchers can capture the diverse experiences of a specific and unique group or tailor their sampling to the groups suggested here in order to achieve a sample comparable to the US Census.

While we focused on the US OCM market in our case study, researchers interested in using non-US OCM samples should be aware that they may differ demographically, and provide statistically significant scale parameter estimates, from consumer panels and US OCM samples [12]. Regardless, researchers should carefully consider how their sample compares to their intended underlying population in order to make valid inferences or generalizations about their results.

### Strengths and Limitations

The differences observed between sampling frames may be due to biases present within each panel. Nonresponse bias occurs when respondents choose to not participate in the survey, which makes it more difficult to draw inferences on the population estimates within the sample. Due to the politicized nature of climate change, and the low compensation rates, respondents may choose not to participate, and thus, contribute to coverage bias by not being represented within the sampling frame. However, we did try to provide a compensation rate that is higher than normal within MTurk ($2 vs. $0.05–$1.00 recommended). On the other side, participants interested in the topic area may be more likely to participate and this would contribute to selection bias in the sample. To address this, we changed the listing for the survey within MTurk, and found no significant differences between these recruitment waves.

Researchers should consider the potential selection bias of internet-based methods which might be influenced by a variety of socioeconomic attributes of the respondents. While this is less of a concern in this study (since both are internet-based), researchers should still limit their inferences when generalizing to the underlying population unless they intend to use sampling weights and qualifications to achieve a nationally representative sample. Additionally, the use of convenience samples, such as OCMs and consumer panels, increases the risk of these biases within a sample. We limited the effect of this bias in our study design by providing the surveys in waves in MTurk and assessing their impact and by including attention check questions to reduce the probability of bots or automated responses.

The effect of timing of the surveys could have been impacted by the COVID-19 pandemic. Ipsos was completed in April 2020, whereas MTurk was completed in July and August 2020. The collective trauma of the COVID-19 pandemic, especially during April–July 2020, may have contributed to the higher proportion of Alarmed and Concerned in the MTurk sample. However, we did compare MTurk’s SASSY segments to the 2019 Ipsos national estimates, and MTurk still had a larger portion of Alarmed and Concerned than Ipsos (Alarmed MTurk, (Ipsos): 34% (31%), Concerned: 32% (26%), Cautious: 19% (16%), Disengaged: 1% (7%), Doubtful: 8% (10%), and Dismissive 6% (10%)) [18].

## 5. Conclusions

Through using SASSY, researchers will be able to more comprehensively understand public opinion on climate change, which can be used for a range of climate change initiatives such as communications, messaging, and other research efforts. While non-probability sampling may have risks, the risks can be managed and accounted for in a public health researcher’s methods and sampling. We encourage public health researchers to utilize qualifications and sampling weights when using consumer panels or OCMs in their research if they intend to achieve a more nationally representative population. However, even without using premium qualifications, we achieved a similar distribution of SASSY segments. Through using SASSY and appropriate sampling techniques, researchers can understand public opinion about climate change in their sample.

## Figures and Tables

**Table 1:** Demographic similarities and differences across US Census, Ipsos, and MTurk samples.

	US Census	Ipsos, April 2020 *n* = 1029	MTurk, July 2020 *n* = 504	*p* (Census vs. Ipsos)	*p* (Census vs. MTurk)	*p* (Ipsos vs. MTurk)
Gender				**0.437**	**0.697**	**0.900**
Male	159,028 (49.0)	517 (50.2)	249 (49.4)			
Female	165,328 (51.0)	512 (49.8)	250 (49.6)			
Age category				**<0.001**	**<0.001**	**<0.001**
Millennials (1981–1996)	66,652 (20.5)	222 (21.6)	258 (51.2)			
Generation X (1965–1980)	60,284 (18.6)	268 (26.0)	173 (34.3)			
Baby Boomers (1946–1964)	73,242 (22.6)	421 (40.9)	72 (14.3)			
Silent (1928–1945)	21,300 (6.6)	81 (7.9)	1 (0.2)			
Greatest (Before 1928)	0 (0.0)	2 (0.2)	0 (0.0)			
Education				**<0.001**	**<0.001**	**<0.001**
Less than high school	26,559 (10.6)	56 (5.4)	3 (0.6)			
High school	70,947 (28.3)	250 (24.3)	60 (11.9)			
Some college	69,577 (27.8)	296 (28.8)	162 (32.1)			
Bachelor’s degree or higher	83,478 (33.3)	427 (41.5)	279 (55.4)			
Income				**<0.001**	**<0.001**	**<0.001**
<$25 K	21,864 (17.0)	98 (9.5)	114 (22.6)			
$25 K–<$50 K	25,755 (20.1)	177 (17.2)	116 (23.0)			
$50 K–<$75 K	21,242 (16.5)	180 (17.5)	149 (29.6)			
$75 K–<$100 K	15,804 (12.3)	130 (12.6)	49 (9.7)			
$100 K–<$125 K	12,114 (9.4)	132 (12.8)	56 (11.1)			
$125 K+	31,674 (24.7)	292 (28.4)	20 (4.0)			
Race/ethnicity ^1^				**<0.001**	**<0.001**	**<0.001**
White, Non-Hispanic	195,060 (76.3)	758 (73.7)	390 (77.4)			
Black, Non-Hispanic	43,464 (13.4)	88 (8.6)	37 (7.3)			
Hispanic	60,095 (18.5)	115 (11.2)	27 (5.4)			
2 + races, Non-Hispanic	9082 (2.8)	27 (2.6)	1 (0.2)			
Other, Non-Hispanic	24,002 (7.4)	41 (4.0)	49 (9.7)			
Region				**0.002**	**<0.001**	**<0.001**
Northeast	22,031 (17.2)	208 (20.2)	116 (23.0)			
Midwest	27,757 (21.6)	244 (23.7)	104 (20.6)			
South	49,486 (38.5)	341 (33.1)	104 (20.6)			
West	29,177 (22.7)	236 (22.9)	179 (35.5)			
SASSY audience segment				--	--	**<0.001**
Alarmed	--	268 (26.0)	172 (34.1)			
Concerned	--	288 (28.0)	161 (31.9)			
Cautious	--	206 (20.0)	93 (18.5)			
Disengaged	--	72 (7.0)	3 (0.6)			
Doubtful	--	113 (11.0)	41 (8.1)			
Dismissive	--	72 (7.0)	30 (6.0)			

^1^ Race and ethnicity categories for US Census data do not add up to 100% because the categories are not mutually exclusive. Note: bolded values are significant at α = 0.05.

**Table 2:** Demographic similarities and differences across SASSY Segment in the MTurk sample, *n* = 504.

	Alarmed *n* (%)	Concerned *n* (%)	Cautious *n* (%)	Doubtful *n* (%)	Dismissive *n* (%)	*p*-Value
Gender						0.378
Male	77 (31.2)	79 (32.0)	50 (20.2)	22 (8.9)	19 (7.7)	
Female	92 (37.6)	80 (32.7)	43 (17.6)	19 (7.8)	11 (4.5)	
Age Category						**0.005**
Millennials (1981–1996)	80 (31.5)	97 (38.2)	54 (21.3)	15 (5.9)	8 (3.2)	
Generation X (1965–1980)	66 (38.4)	42 (24.4)	30 (17.4)	17 (9.9)	17 (9.9)	
Baby Boomers or older (1946–1964)	26 (36.6)	22 (31.0)	9 (12.7)	9 (12.7)	5 (7.0)	
Education						0.074
Some college or less	50 (32.5)	48 (31.2)	28 (18.2)	21 (13.6)	7 (4.6)	
Associate/Bachelor or more	122 (35.6)	113 (32.9)	65 (18.9)	20 (5.8)	23 (6.7)	
Total household income (in 1000 s)						0.050
Less than $30	41 (36.9)	34 (30.6)	15 (13.5)	13 (11.7)	8 (7.2)	
$30–99	97 (31.3)	108 (34.8)	67 (21.6)	24 (7.7)	14 (4.5)	
$100 or more	34 (44.7)	19 (25.0)	11 (14.5)	4 (5.3)	8 (10.5)	
Race/ethnicity						0.741
White, Non-Hispanic	129 (33.4)	128 (33.2)	73 (18.9)	33 (8.6)	23 (5.9)	
Black, Non-Hispanic	14 (38.9)	8 (22.2)	8 (22.2)	3 (8.3)	3 (8.3)	
Hispanic	8 (29.6)	10 (37.0)	3 (11.1)	4 (14.8)	2 (7.4)	
Other, Non-Hispanic	21 (43.8)	15 (31.3)	9 (18.8)	1 (2.1)	2 (4.2)	
Region						0.860
West	46 (40.4)	34 (29.8)	18 (15.8)	10 (8.8)	6 (5.3)	
Northeast	39 (37.9)	34 (33.0)	20 (19.4)	6 (5.8)	4 (3.9)	
Midwest	29 (28.2)	38 (36.9)	19 (18.5)	9 (8.7)	8 (7.8)	
South	58 (32.9)	55 (31.3)	36 (20.5)	15 (8.5)	12 (6.8)	

Note: bolded values are significant at α = 0.05.

## Data Availability

Data and supporting materials in Stata 16 will be made available upon reasonable request from the corresponding author.

## Supplementary Materials

The following are available online at https://www.mdpi.com/article/10.3390/ijerph19148320/s1, Table S1: Survey questions used in the analysis; Table S2: Normalization of survey demographics to GMU survey design.
