# Supplementary material for: Global Warming’s Six MTurks: A Secondary Analysis of a US-Based Online Crowdsourcing Market
Source: Int J Environ Res Public Health. 2022 Jul 7;19(14):8320. doi: 10.3390/ijerph19148320 (PMC9323866; doi:10.3390/ijerph19148320)
Supplement: Supplementary file 1 [file ijerph-19-08320-s001.zip › ijerph-1712222-supplementary.pdf]

**Table S1.** Survey Questions Used in this Analysis.

| Variable | Question | Answer options |
|---|---|---|
| Belief4 | How much do you think global warming will harm you personally? | Not at all (1)<br>Only a little (2)<br>A moderate amount (3)<br>A great deal (4)<br>Don't know (0) |
| Belief5 | How much do you think global warming will harm future generations? | Not at all (1)<br>Only a little (2)<br>A moderate amount (3)<br>A great deal (4)<br>Don't know (0) |
| Inv15 | How worried are you about global warming? | Very worried (4)<br>Somewhat worried (3)<br>Not very worried (2)<br>Not at all worried (1) |
| Inv18 | How important is the issue of global warming to you personally? | Not at all important (1)<br>Not too important (2)<br>Somewhat important (3)<br>Very important (4)<br>Extremely important (5) |
| sex | What is your gender identity? | Male (1)<br>Female (0)<br>Transgender Male (female to male) (2)<br>Transgender Female (male to female) (3)<br>Non-binary or Gender neutral (4)<br>Prefer to self describe: (5) |
| race | Which of these groups would you say best describes your race and ethnicity? Mark all that apply. | White or Caucasian (1) Black or African American (2) American Indian or Alaska Native (3) Asian or Pacific Islander (4) Hispanic or Latino (5) Prefer to self describe: |
| state | In which state do you currently reside? | List of 52 states |
| income | What is your household's total income (in previous year, 2019) before taxes? | Less than $10,000 (1)<br>$10,000 to $19,999 (2)<br>$20,000 to $29,999 (3)<br>$30,000 to $39,999 (4)<br>$40,000 to $49,999 (5)<br>$50,000 to $59,999 (6)<br>$60,000 to $69,999 (7)<br>$70,000 to $79,999 (8)<br>$80,000 to $89,999 (9)<br>$90,000 to $99,999 (10)<br>$100,000 to $149,999 (11)<br>$150,000 or more (12) |

| | | |
|---|---|---|
| education | What is the highest level of school you have completed or the highest degree you have received? | Less than high school degree (1)<br>High school graduate (high school diploma or equivalent including GED) (2)<br>Some college but no degree (3)<br>Associate degree in college (2-year) (4)<br>Bachelor's degree in college (4-year) (5)<br>Master's degree (6)<br>Doctoral degree (7)<br>Professional degree (JD, MD) (8) |

**Table S2.** Normalization of our survey demographics to GMUs.

| Variable Normalization | MTurk Variables | Ipsos Categories | US Census Categories (ACS 2019, 2010 Census) |
|---|---|---|---|
| Gender identity to Sex | Male, Transgender Male | Male | Male |
| | Female, Transgender Female | Female | Female |
| | Non-Binary or Gender Neutral, or Prefer to self-describe | Other (not originally included in GMU survey) | N/A |
| Age to Generation Group | Age <23 | iGen (1997 -) | Sum of age groups 5-24 years |
| | 24 to 39 | Millenials (1981 – 1996) | Sum of age groups 25-39 years |
| | 40 to 55 | Generation X (1965 – 1980) | Age group 40-54 years |
| | 56 to 74 | Baby Boomers (1946 – 1964) | Sum of age groups 55-74 years |
| | 75 to 92 | Silent (1928 – 1945) | Sum of age groups 75-84, 85 years and over |
| | No participants | Greatest (Before 1928) | N/A |
| Education level to Education Group | Less than high school degree | Less than high school | 1st - 4th grade<br>5th - 6th grade<br>7th - 8th grade<br>9th grade<br>10th grade<br>11th grade |
| | High school graduate (high school diploma or equivalent including GED) | High school | High school graduate |
| | Some college but no degree,<br>Associate degree in college (2-year) | Some College | Some college, no degree, Associate's degree, occupational |

| | | | |
|---|---|---|---|
| | | | Associate's degree, academic |
| | Bachelor's degree in college (4-year), Master's degree, Doctoral degree, Professional degree (JD, MD) | Bachelor’s degree or higher | Bachelor's degree<br>Master's degree<br>Professional degree<br>Doctoral degree |
| Income level to Income Group | Less than $10,000, $10,000 to $19,999, $20,000 to $29,999 | <$25K | Under $5,000<br>$5,000 to $9,999<br>$10,000 to $14,999<br>$15,000 to $19,999<br>$20,000 to $24,999 |
| | $30,000 to $39,999, $40,000 to $49,999 | $25K - <$50K | $25,000 to $29,999<br>$30,000 to $34,999<br>$35,000 to $39,999<br>$40,000 to $44,999<br>$45,000 to $49,999 |
| | $50,000 to $59,999, $60,000 to $69,999, $70,000 to $79,999 | $50K - <$75K | $50,000 to $54,999<br>$55,000 to $59,999<br>$60,000 to $64,999<br>$65,000 to $69,999<br>$70,000 to $74,999 |
| | $80,000 to $89,999, $90,000 to $99,999 | $75K - <$100K | $75,000 to $79,999<br>$80,000 to $84,999<br>$85,000 to $89,999<br>$90,000 to $94,999<br>$95,000 to $99,999 |
| | $100,000 to $149,999 | $100K - <$1250K | $100,000 to $104,999<br>$105,000 to $109,999<br>$110,000 to $114,999<br>$115,000 to $119,999<br>$120,000 to $124,999 |
| | $150,000 or more | $125K+ | $125,000 to $129,999<br>$130,000 to $134,999<br>$135,000 to $139,999<br>$140,000 to $144,999<br>$145,000 to $149,999<br>$150,000 to $154,999<br>$155,000 to $159,999<br>$160,000 to $164,999<br>$165,000 to $169,999<br>$170,000 to $174,999<br>$175,000 to $179,999<br>$180,000 to $184,999<br>$185,000 to $189,999<br>$190,000 to $194,999 |

| | | | |
|---|---|---|---|
| | | | \$195,000 to \$199,999<br>\$200,000 and over |
| Race and Ethnicity to Race/Ethnic Group | White, Non-Hispanic | White, Non-Hispanic | White, Non-Hispanic |
| | Black, Non-Hispanic | Black, Non-Hispanic | Black, Non-Hispanic |
| | Hispanic | Hispanic | Hispanic |
| | 2+ Races, Non-Hispanic | 2+ Races, Non-Hispanic | 2+ Races, Non-Hispanic |
| | American Indian or Alaska Native, Asian or Pacific Islander, Prefer to self-describe AND Non-Hispanic | Other, Non-Hispanic | Other, Non-Hispanic |
| State to Region | CT, MI, MA, NH, NJ, NY, PA, RI | Northeast | Northeast |
| | IL, IN, IA, KS, MI, MN, MO, NE, ND, OH, WI | Midwest | Midwest |
| | AL, AK, FL, GA, KT, LO, MA, MS, NC, OK, SC, TN, TX, VI, WV | South | South |
| | AZ, CA, CO, HI, ID, MT, NV, NM, OR, UT, WA | West | West |

Source: U.S. Census Bureau, Current Population Survey, 2020 Annual Social and Economic Supplement (CPS ASEC). Available online: https://www.census.gov/data/tables/time-series/demo/income-poverty/cps-hinc/hinc-01.2019.html (accessed on July 5th, 2022).
